# Percutaneous endoscopic thoracic discectomy via posterolateral approach: A case report of migrated thoracic disc herniation

**DOI:** 10.1097/MD.0000000000017579

**Published:** 2019-10-11

**Authors:** Wei Liu, Liyu Yao, Xingchen Li, Zhisen Tian, Cong Ning, Ming Yan, Yuanyi Wang

**Affiliations:** a Department of Spine Surgery, the First Hospital of Jilin University; b Department of Pediatric Surgery, the First Hospital of Jilin University, Changchun; c Intervertebral Disc Center, the Third Hospital of Henan Province, Zhengzhou; d Department of Spine Surgery, China-Japan Union Hospital of Jilin University, Changchun, P.R. China.

**Keywords:** endoscopic reamer, percutaneous endoscopic thoracic discectomy, percutaneous spine endoscopy, thoracic disc herniation, thoracic stenosis

## Abstract

**Rationale:**

Minimally invasive surgeries for thoracic disc herniation (TDH) evolved rapidly in recent years, and multiple approaches have been put forward. Thoracic discectomy via percutaneous spine endoscopy (PSE) is inadequately documented because of the low prevalence of TDH and the high difficulty of thoracic spine endoscopy techniques. Herein, we present a TDH case who underwent percutaneous endoscopic thoracic discectomy.

**Patient concerns:**

A 28-year-old male suffered backpain and partial paralysis in lower extremities.

**Diagnoses:**

Magnet resonance imaging demonstrated T11-12 TDH, with cranially migrated disc fragment.

**Interventions:**

The patient underwent percutaneous endoscopic thoracic discectomy via posterolateral approach with the assistance of endoscopic reamer in the procedure of foramino-laminaplasty.

**Outcomes:**

The patient's muscle force improved immediately, and the backpain relieved after 5 days post-surgery. In the 6-month follow-up, he had normal muscle force without paresthesia in lower limbs.

**Lessons:**

The innovative design of endoscopic reamer provides effective plasty and access establishment with lower risk and difficulty, which ensures the vision and the operating space of the procedure of decompression. With this technique, the indications of thoracic PSE were broadened to both ventral and dorsal thoracic stenosis.

## 1 Introduction

Percutaneous spine endoscopy (PSE) is a well-accepted minimally invasive surgery (MIS) that made enormous progress along with the evolution of medical industry within the last 2 decades. Date back to 1980s, Kambin et al^[1]^ described the theory of Kambin triangle which allowed surgeons to operate within the specific safe area via percutaneous lateral approach. At the start of 21^st^ century, Yeung et al^[2]^ and Hoogland et al^[3]^ initiated a boost of lumbar PSE by proposing their own approaches and techniques with distinctive concepts, respectively. PSE has become a popular surgery favored by patients and surgeons for its advantages over conventional open surgery including immediate pain relief, less surgical injury, quick recovery, and more simplex anesthesia requirements.^[4,5]^ With the ongoing increase of application, the indications of PSE have as well been broadened. In recent years, various newly designed approaches with matched surgical instruments have been described to manage complicated conditions and targets,^[6,7]^ which endowed a breakthrough that allowed PSE to treat nearly any lumbar disorders.^[8]^ In cervical spine, PSE via anterior or posterior approach are well established and result in excellent outcomes.^[9,10]^

Although not as high as the prevalence of cervical and lumbar disorders, thoracic degeneration is accumulating attention. Thoracic MIS can be categorized in 2 major approaches including thoracoscopic surgery and the variations of microendoscopic surgery. Thoracoscopy discectomy gained popularity in the last decades; however, the demands of patient subset selection and pulmonary and neural complications limited the development of thoracoscopy discectomy.^[11]^ Thoracic PSE discectomy was firstly performed in 1990s with the assistance of other instrument^[12]^; nevertheless, PSE was not solely applied in thoracic spine until 2010s, which was based on great success of lumbar PSE.^[13]^ At present, thoracic PSE is adopted in common thoracic conditions, including thoracic disc herniation (TDH)^[14]^ and ossification of ligamentous flavum (OLF), which are main etiologies of thoracic stenosis.^[15]^ However, because of the late cognition by surgeons, the technique and outcome of thoracic PSE vary greatly. Besides, there is no approach that is efficacious and safe enough to be accepted and applied like TESSYS or YESS technique in lumbar spine.

Herein, we present a case of a TDH patient who underwent thoracic PSE with excellent outcome. This case highlights the safety and efficacy of PSE with endoscopic reamers, which might be a potential standardized MIS strategy for treating TDH.

## 2 Case report

This study was approved by the Ethics Committee and Institutional Review Board of the First Hospital of Jilin University.

A 28-year-old male presented to department of spine surgery with backpain and weakness in lower extremities for 1 year. His lower extremity symptoms worsened over 2 months with rapid progress.

**Figure 1:**
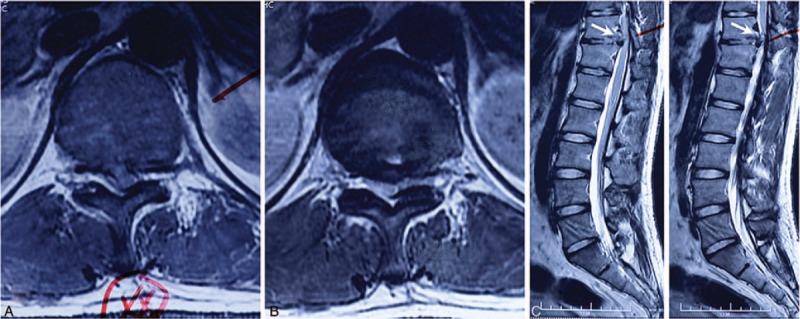
Preoperation magnetic resonance imaging revealed disc herniation on T11-12. Horizontal view (A and B) displayed secondary thoracic stenosis induced by herniated disc fragment; sagittal view to the left (C and D) demonstrated that cranially migrated disc fragment compressed dura sac (arrow).

On physical examination, the patient showed stable vital features. He has moderate back tenderness, and restriction of back motion range. The patient's nervous system examination results included muscle force loss of lower extremities, which was more severe on his left lower limb (3/5). He as well presented sensory changes below the inguinal level. His Babinski sign was positive. The patient scored 5 on the visual analog scale (VAS) and scored 69 according to the Oswestry disability index (ODI). Magnetic resonance imaging (MRI) demonstrated TDH on the level of T11-12 (Fig. 1), and sagittal view revealed a cranially migrated herniated disc fragment in the left side of spinal canal, which caused compression to the corresponding dura sac (Fig. 1C and D). According to the history above, the patient was diagonosed as having TDH (T11-12), secondary thoracic stenosis, and partial paralysis on lower extremities.

**Figure 2:**
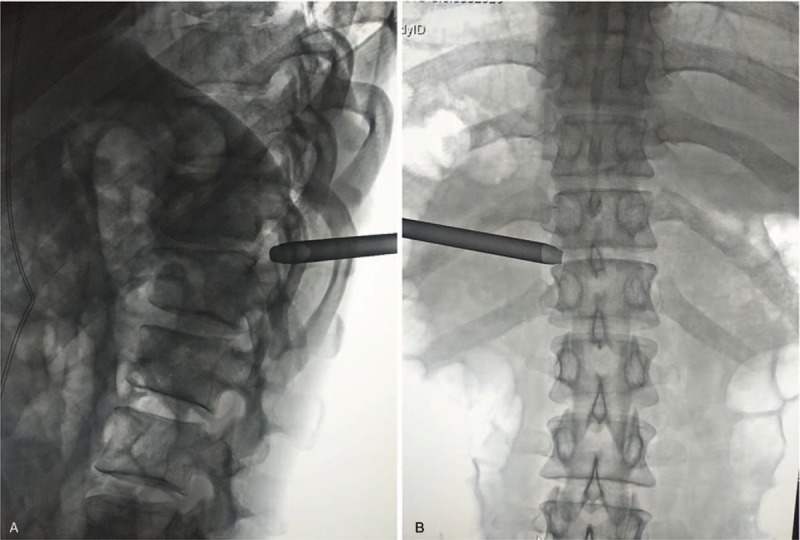
Intraoperation C-arm fluoroscopy displayed the location of the reamer cannula. The LT view showed that the distal end of the reamer cannula was anchored upon the cortex of superior articular process body of T12 (A); on the AP view, the cannula tip was on the outside margin of the intravertebral foramina (B). AP = antero-posterior, LT = lateral.

**Figure 3:**
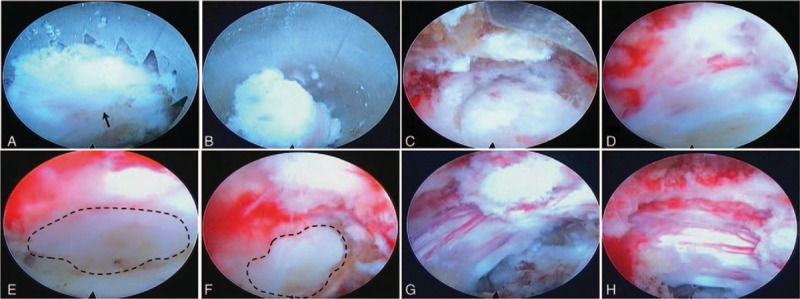
Intraoperation endoscopic views. After identifying the facet joint (A, arrow), the reamer was operated manually to remove the corresponding part of superior articular process (B). When the procedure of foramino-laminaplasty was completed, part of the facet and laminar was resected (C and D). When the dura sac was adequately exposed, the compressive migrated disc fragment was in succession identified (E and F, outline) and removed. After thorough decompression, the dura sac regained autonomous pulse and abundant surface blood flow (g & h).

For the operation, the patient was positioned in prone position with support of silicon pads. The midline was marked over the spinous processes of T11-12 under C-arm fluoroscopy. The puncture point was selected 7 cm to the left of the midline between the ribs, which was measured on the MRI and applied according to the scale. After diazepam intramuscular injection and routine sterilizing and draping, local anesthesia was administrated around the puncture with 1% lidocaine in the order of subcutaneous, facia, and periosteum. After confirming the puncture needle reached the facet of T11-12 with the aid of C-arm fluoroscopy, a skin incision was made and a guide wire was inserted through the puncture needle. Along the guide wire, the dilators were inserted to expand paraspinal soft tissue. Then, the PSE endoscope with matched endoscopic reamer was inserted via the reamer cannula, which was placed along the guide wire and whose location was ensured by C-arm fluoroscopy (Fig. 2). The joint of superior/inferior articular process (SAP/IAP) was identified under endoscope (Fig. 3A). Monitored in video, the endoscopic reamer was rotated manually and the tip of SAP was removed (Fig. 3B). With the same method, the procedure of foramino-laminaplasty was completed, which removed the facet and the left part of T11 laminar (Fig. 3C and D). Then, the reamer and its cannula were replaced by working cannula and the decompression procedure was performed using fine forceps. After exposing the dura by removing ligamentous flavum, the compression in ventral side of dura sac including the cranially migrated disc fragment was identified (Fig. 3E and F) and resected. During discectomy, the patient reported backpain when the dura sac was excessively pushed, and sensory improvement as the decompression proceeded. The decompression was completed when autonomous pulse of dura sac and the blood flow were regained without suspicious compression (Fig. 3G and H). Totally, the operation time was 2.5 hours and the blood loss was <50 mL.

**Figure 4:**
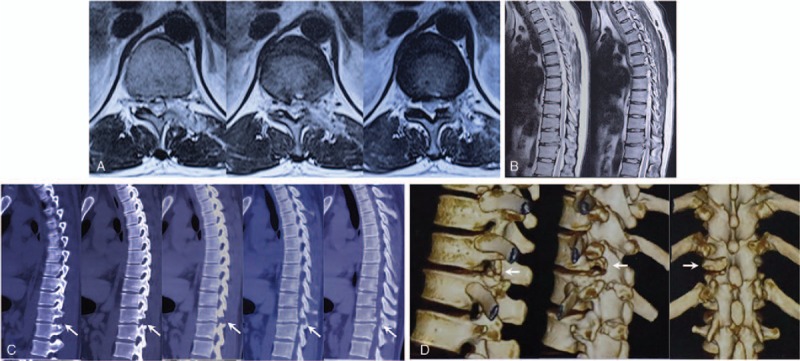
Post-operation imaging revealed satisfying decompression on T11–12. Magnetic resonance imaging demonstrated restored spinal canal and postoperative change of the disc and laminar (A and B). Computed tomography scan displayed that part of the facet and laminar was resected, which provided adequate vision and space for decompression (C and D, arrow).

The patient's muscle force increased to 4/5 immediately after surgery and his backpain relieved (VAS = 1) in 5 days post-surgery, only slight residual numbness on his left lower limb was reported. Postoperative MRI indicated thorough decompression in the spinal canal (Fig. 4A), and computed tomography (CT) scan displayed ideal plasty range (Fig. 4C). In 1-month follow-up, the patient's muscle force was completely recovered and the residual numbness area decreased, and the ODI was 14. In 6-month follow-up, he had normal muscle force without paresthesia (VAS = 0, ODI = 9). No stability symptom was presented since discharge to 6-month follow-up.

## 3 Discussion

As the booming development of PSE within the last 2 decades, the indication of PSE has been significantly broadened. Lumbar discectomy is the first PSE technique that widely accepted by spine surgeons for its safety and efficacy proved by worldwide clinical practice. To date, the application of PSE in almost all kinds of complicated LDH and lumbar stenosis were well documented in literature with a wealth of experience concluded by clinicians.^[16–18]^ Compared with lumbar PSE, cervical PSE requires selected indications, more specific surgical instruments and more effective anesthesia in both anterior and posterior approaches.^[19]^

Thoracic spine has less mobility than cervical spine and less load pressure than lumbar spine, as a result, symptomatic thoracic stenosis has the lowest incidence. Although there is a considerable rate of asymptomatic TDH (11%–37%),^[20]^ TDH requires surgical treatment accounts only 0.15%to 1.8% of all surgically treated disc herniations.^[21]^ With the development of imaging examination, the diagnosis of thoracic stenosis has increased, surgical techniques with less invasion and higher safety are desiderated demanded. The primary goal of thoracic PSE is to execute decompression as conventional open surgeries with reduced muscular injury and bleeding.^[11]^ Concurrently, plenty of surgical techniques have been put forward by surgeons, Jho et al^[12]^ described endoscope-assistant discectomy for TDH via transpedicular approach, whose working channel building required aid of microscope and drills. Choi et al^[22]^ reported thoracic discectomy using tubular retractor via oblique approach, although these procedures guaranteed the vision by adopting highspeed drills, only partial or none PSE technique were employed. Thoracic PSE via transforaminal approach is a reasonable thoracic PSE technique for treating TDH, and different procedures mainly distinct in the foraminoplasty details.^[14,23]^ These literatures have proved that PSE system was able to perform thoracic discectomy without assistant of other techniques or instruments. However, complications appeared along the evolution of thoracic PSE. The most commonly reported complication is cerebral fluid (CF) leak caused by dura injury,^[13,15]^ which also frequently occurs in lumbar and cervical PSE surgeries. Besides, complications that frequently occur in other approaches such as recurrence and residual disc were not fully reported, the reason might be attributed to that thoracic PSE is not popularized. Although the safety and surgical outcome have been improved in recent years,^[21]^ the sole-application of PSE in thoracic spine has yet been adequately documented.

In current case, the patient had migrated TDH, and it required expanded exposure range to ensure motion range and angle of endoscope device. Hence, we applied endoscopic reamers in the plasty step for TDH, which has not been reported. The innovative design of endoscopic reamer enables the plasty of several parts of spine with ease and safety. It allows surgeons to perform plasty and bony decompression under video surveillance, which have significantly increased the safety and accuracy of the reamers. Despite of skilled surgeons can perform relatively safe leminar or foraminal plasty by using common reamers monitored by C-arm fluoroscopy in lumbar and thoracic discectomy,^[3,15,24]^ these techniques to great extent rely on the operation experience including location estimate under C-arm and sensitive hand feeling, which makes PSE a difficult surgery to learn.^[25]^ Therefore, the creative innovations like navigation system and endoscopic reamer are meaningful to surgeons.^[26]^

During the surgery, the plasty procedure removed the tip of SAP and part of IAP body with endoscopic reamer, and the exposure range was as large as conventional facet fenestration and provided adequate decompression vision and motion range of endoscope device. Some structures including ligamentum flavum, dorsal and lateral dura sac, posterior longitudinal ligament and intervertebral disc can be clearly observed and easily reached under the endoscope vision. As a result, this approach allows surgeons to handle the ventral compression like TDH and the compression on the dorsal side like OLF. Zhao et al^[15]^ described similar procedure for treatment of ligamentum flavum ossification, and reported with satisfied outcome with few surgical complications, which supported the high applicability of our approach. The patient's TDH was in the left-median part of spinal canal; therefore, we adopted lateral approach and underwent foraminoplasty to ensure the extension toward median line. Thoracic spinal cord is vulnerable against mechanical pressure. In lumbar PSE, the working cannula can easily reach the median line by shoving and pushing away the traveling nerve root^[24]^; different from lumbar PSE, aggressive traction in thoracic spinal cord may cause serious neural complications because of the traction bring up direct pressure on the dura sac in thoracic instead of the nerve root in lumbar. As a result, we as well adopted the posterior approach by partially removing the laminar and IAP body to prevent double-sided pressure from ventral side and dorsal side while we extending the working cannula toward the midline of spinal canal, and we merely performed push-away by 2 to 3 mm when decompressing the ventral TDH to avoid excessive dura sac traction, and the traction-genic back pain was another indicator of excessive shoving which was closely concerned during the procedure of decompression.

Through this case of TDH, we present our endoscopic foramino-laminaplasty technique and discectomy under PSE via thoracic posterolateral approach with satisfying clinical outcome. This is the first time that thoracic PSE with endoscopic reamer is reported in literature. The application of endoscopic reamer allows PSE to jump out of the circle of simplex posterior or lateral approach and laminar or foraminoplasty with elevated safety, and provides dynamic support to targeting decompression. It enables surgeons to expand or narrow the plasty range according to the location and volume of decompression target, which can reduce unnecessary injury and conform to the minimally invasive concepts. As a novel application, there are several concerns that ought to be mentioned. First, we proposed a posterolateral approach as an effective treatment for soft TDH and OLF. Second, the exposure range can be customized according to the necessity of decompression vision with no affection to stability when part of the facet and laminar were properly removed^[15]^; nonetheless, over-resecting may lead to instability and posterior pressure of dura sac. Therefore, preoperative designation is extremely important. Third, this method has not been adopted in upper thoracic spine. Theoretically, the posterior part of our approach is able to be conducted normally, which ensures the decompression of OLF. Whereas, the lateral part might be blocked by scapula, who interferes the foraminoplasty and the ventral decompression. As a result, our approach in upper thoracic spine demands further modifications and tests. Fourth, CF leak is the most common complication in thoracic spine surgeries. In conventional open surgery, several parts of the dura can be sutured, whereas there is no instrument that designed for dura suture under PSE. Hence, tissue adhesion ought to be handled in the last step with caution. According to our experience, operation temporarily suspension is routine when CF leak occurs, and when and whether to continue the procedure depend on the patient's symptoms and the remaining work. Additionally, postoperation drainage is essential for CF leak.

## 4 Conclusion

Collectively, we presented a case with migrated TDH, and we underwent thoracic PSE using endoscopic reamers and acquired excellent postoperation outcomes with few further symptoms in follow-up. Our approach is a safe, effective procedure for treating TDH, and it has expanded the application of PSE in the entire spine.

## Author contributions

**Funding acquisition:** Yuanyi Wang.

**Investigation:** Wei Liu, Ming Yan.

**Resources:** Wei Liu, Xingchen Li.

**Software:** Liyu Yao.

**Visualization:** Liyu Yao, Zhisen Tian.

**Writing – original draft:** Cong Ning, Yuanyi Wang.

**Writing – review & editing:** Yuanyi Wang.

Yuanyi Wang orcid: 0000-0002-9191-3782.
